# The impact of incomplete registration on survival rate of children with very rare tumors

**DOI:** 10.1038/s41598-021-93670-2

**Published:** 2021-07-07

**Authors:** Jelena Rascon, Lukas Salasevicius, Giedre Rutkauskiene, Ewa Bien, Ieva Vincerzevskiene

**Affiliations:** 1 Center for Pediatric Oncology and Hematology, Vilnius University Hospital Santaros Klinikos, Santariškių 4, 08406 Vilnius, Lithuania; 2 Faculty of Medicine, Vilnius University, M. K. Čiurlionio 21/27, 03101 Vilnius, Lithuania; 3 Lithuanian University of Health Sciences, A. Mickevičiaus 9, 44307 Kaunas, Lithuania; 4 Department of Pediatrics, Hematology and Oncology, Medical University of Gdansk, 7 Debinki Street; 80-211, Gdansk, Poland; 5 National Cancer Institute, Cancer Registry, Santariškių 1, 08660 Vilnius, Lithuania

**Keywords:** Diseases, Medical research, Oncology

## Abstract

Pediatric very rare tumors (VRTs) represent a heterogeneous subset of childhood cancers, with reliable survival estimates depending dramatically on each (un)registered case. The current study aimed to evaluate the number of VRTs among Lithuanian children, to assess the impact of the registration status on survival rates and to track changes in treatment outcomes over the 16-year study period. We performed a population-based retrospective study across children below 18 years old diagnosed with VRTs in Lithuania between the years 2000 and 2015. The identified cases were cross-checked with the Lithuanian Cancer Registry—a population-based epidemiology cancer registry—for the fact of registration and survival status. The overall survival was calculated in relation to the registration status and treatment period. Thirty-seven children with VRTs were identified within the defined time frame. Six of them (16.2%) were not reported to the Lithuanian Cancer Registry at diagnosis. The probability of overall survival at 5 years (OS_5y_) differed significantly between the registered (n = 31) and unregistered (n = 6) cohorts: 51.6% versus 100%, respectively (*p* = 0.049). A 5-year survival estimate for children diagnosed with a VRT at the age of 0–14 years differed by 10 percentage points according to the registration completeness: 52.1% calculated for the entire cohort versus 42.1% for registered patients only. The OS_5y_ has not improved over the analyzed period: 61.1% in 2000–2007 versus 57.9% in 2008–2015 (*p* = 0.805). The survival continued to decline beyond 5 years post-diagnosis due to late cancer-related adverse events: 59.5% of patients were alive at 5 years as compared to 44.3% at 10 years. The OS_5y_ of children affected by VRT was lower than in more common childhood cancers. The survival rate of the unregistered patients may lead to misinterpretation of treatment outcomes. Meticulous registration of VRTs is crucial for correct evaluation of treatment outcomes, especially across small countries with few cases.

## Introduction

Malignant tumors in children are very rare: a recently calculated incidence of pediatric cancer was 155.8 cases per million children between the ages of 0 to 19 years^1^. Although infrequent, the vast majority of pediatric cancers can be treated in international clinical trials or following treatment guidelines developed by expert groups. Despite the well-elaborated management strategies for the most common childhood malignancies, childhood cancer remains the second leading cause of disease-related mortality among children in developed countries^2^.

Pediatric very rare tumors (VRTs) represent a particular subset of childhood cancers, comprising approximately 9 to 11% of all malignancies occurring in children and adolescents below the age of 20, 75% of them being diagnosed between the ages of 15 to 19 years^3–5^. The European Cooperative Study Group for Pediatric Rare Tumors (EXPeRT) defined a VRT as any solid malignancy or borderline tumor that has an incidence rate of ≤ 2 per million per year and/or is not considered for treatment in clinical trials^6^. This definition was adopted by the EXPeRT members to re-examine a list of malignant entities considered as pediatric VRT under umbrella of the Joint Action in Rare Cancers project^5^. In clinical practice, VRTs pose a diagnostic and therapeutic challenge due to their extreme rarity and lack of uniform treatment guidelines and ongoing clinical trials.

The overall survival rates of childhood cancer differ across European countries. Pooled data provided by the European cancer registries for the EUROCARE5 study demonstrated that survival rates of children treated between 2000 and 2007 in Eastern Europe were lower by 20–30% than in other European countries^7–9^. In particular, the five-year survival rates in Lithuania were approximately 10–20% lower as compared to the European average. A quality study evaluating completeness of the registration in Estonian Cancer Registry demonstrated that under-reporting of pediatric malignancies can decrease the incidence of childhood cancer and survival estimates^10^. Our previous pilot study focused on the tumors of the central nervous system (CNS) revealed that up to 27% of cases were missing in the Lithuanian Cancer Registry (LCR)^11^. Based on this observation, we initiated the current population-based study that aimed to evaluate the number of VRTs in Lithuanian children and to verify their registration status in the LCR as well as its potential impact on survival rates.

## Patients and methods

### Study population and design

We performed a retrospective population-based study including Lithuanian children below 18 years of age diagnosed with VRTs between the years 2000 and 2015. A VRT was defined as described and recently reviewed by the International EXPeRT Cooperative Group (see above)^5,6^. Only malignant entities (ICD-10-AM codes C00-C96) were selected for evaluation. Pediatric age was defined as below 18 years at diagnosis.

All Lithuanian children with cancer are treated at two university hospitals: Vilnius University Hospital Santaros Klinikos (VUHSK) and Lithuanian University of Health Sciences Kaunas Clinics (LUHSKC). The institutional databases of both pediatric oncology centers were reviewed to identify VRTs as described above. To verify the fact of registration, the retrieved cases were cross-checked with the LCR using personal identification code unique for each individual. Each patient’s vital status (and the date of death where appropriate) were updated on 31st of December 2019 using a linkage with the national population registry. Patients lost for follow-up were considered alive and censored at the last available contact date.

LCR is a nationwide population-based cancer registry covering the whole country^12^. Health care providers have a legal obligation to report each new cancer case (ICD-10-AM codes: malignant neoplasms C00–C96, in situ neoplasms D00–D09, benign neoplasm of meninges, brain and other parts of CNS D32–D33, neoplasms of unspecified behavior D39.1, D42–D43, D45–D47). Unlike many cancer registries, the LCR does not include non-malignant tumors of pituitary and pineal glands (ICD-10-AM code D35). LCR collects personal and demographic data as well as disease-related information (cancer site, date of diagnosis, method of cancer verification) and vital status (if appropriate—date and cause of death) of all cancer patients in Lithuania including children. The collected data set (ICD-10-AM codes, personal and disease-related information) is embedded in the national legislation that governs LCR activities.

Baseline patients’ characteristics were collected electronically through institutional databases, or, when needed, manually through paper records. To assess treatment outcomes a probability of overall survival at 5 years from diagnosis (OS_5y_) was considered as a primary endpoint. Survival at 1 and 10 years were assessed as secondary endpoints. For international comparison, survival estimates were additionally calculated for the age group of 0–14 years at diagnosis. The overall survival was compared between the registered and unregistered patients. To evaluate changes in survival rates over the 16-year study period, the entire cohort was split into two groups according to the treatment period: between 2000 and 2007 versus between 2008 and 2015.

### Statistical analysis

Descriptive statistics methods were used for abnormally distributed continuous and categorical variables. A probability of overall survival at 5 and 10 years were calculated using Kaplan–Meier estimation method. The difference between compared groups was compared using log-rank test. Death of any cause (tumor relapse or progression, treatment-related toxicity and second cancer) was defined as an event. The results were considered statistically significant when *p* value was < 0.05. The statistical analysis was performed using IBM SPSS Statistics 27.0 and STATA IC 11.0 (StataCorp LP) softwares.

### Ethics approval and consent to participate

All procedures performed in studies involving human participants were in accordance with the ethical standards of the institutional and national research committee and with the 1964 Helsinki declaration and its later amendments or comparable ethical standards. The study was approved by the Vilnius Regional Committee of Biomedical Research (Approval No. 2019/10-1155-646) and Kaunas Regional Biomedical Research Ethics Committee (Approval No. BE-2-86). An informed consent was obtained from individual participants subject they are alive and followed up. A written informed consent was obtained from parents or guardians for participants under 16 years old. A waiver of informed consent was granted for patients deceased or lost for follow-up by the Vilnius Regional Committee of Biomedical Research (Approval No. 2019/10-1155-646).

## Results

### Patients’ characteristics

**Figure 1:**
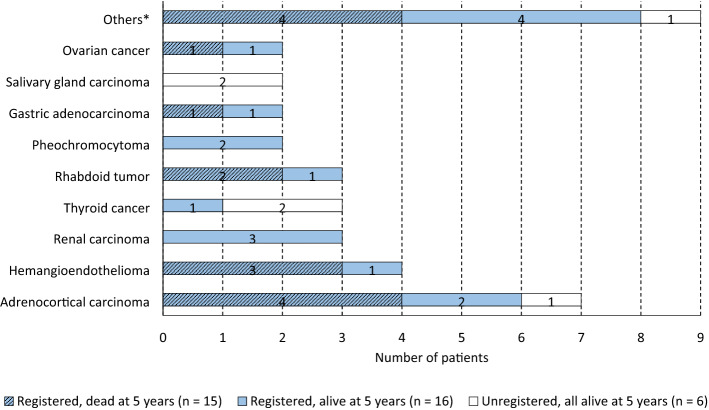
Distribution of the analyzed VRTs and survival status at 5 years across tumor types with regard to registration status. *The group “Others” comprised 9 single VRT types. Eight cases were registered: 4 patients (colorectal carcinoma, desmoplastic small round cell tumor, intestinal neuroendocrine carcinoma, follicular dendritic cell sarcoma) did not survive by 5 years, the other 4 (lip carcinoma, melanoma, pancreatic carcinoma, lung carcinoma) remained alive. One unregistered patient (uterine adenosarcoma) was alive at the same time point.

In total, 37 children were treated for VRTs between the years 2000 and 2015 in Lithuania. Over 16-year study period, a median of 1.5 new cases (ranging from 0 to 6) was diagnosed per year. Baseline characteristics of the enrolled patients are summarized in Table 1. The median age at diagnosis was 12 years (range 0–17, IQR [6.5–15.5]). Children diagnosed and treated at VUHSK tended to be younger than peers at LUHSKC (the median age at diagnosis was 10.5 years (range 0–17, IQR [2.8–14.3] and 15.0 years (range 0–17, IQR [9.0–17.0], respectively). Otherwise, baseline characteristic between two pediatric oncology center cohorts did not differ (Supplementary Table S1). A slight female predominance (n = 21; 57%) was revealed. Overall, 18 histologic types of VRTs were identified (Fig. 1) with adrenocortical carcinoma being the most frequent (n = 7; 18.9%), followed by hemangioendothelioma (n = 4; 10.8%). Renal and thyroid carcinoma as well as rhabdoid tumor accounted for 3 (8.1%) cases each. There were two cases of pheochromocytoma, gastric adenocarcinoma, salivary gland carcinoma, and ovarian cancer (5.4% each) and 9 single cases of various tumor types (Fig. 1).

**Table 1:** Baseline characteristics of the study patients (n = 37).

UPN	Gender	Age at dia-gnosis, years	Year of dia-gnosis	Cancer type	Cancer predispo-sition syndrome	Event	Out-come	Follow-up (years)	Registration at LCR	Treating center
1	F	14	2000	Hemangioendothelioma of atrium (L)	NS	Progress	Died	1.1	Registered	VUHSK
2	M	10	2000	Pheochromocytoma (L)	VHL	CR	Alive	19.3	Registered	VUHSK
3	F	11	2001	Lung cancer (L)	NS	CR	Alive	18.1	Registered	VUHSK
4	F	2	2002	Liver angiosarcoma (L)	NS	Progress	Died	1.3	Registered	VUHSK
5	M	16	2005	Adrenocortical carcinoma (A)	NS	Progress	Died	0.6	Registered	VUHSK
6	F	17	2005	Uterine adenosarcoma (L)	NS	2ndCa	Died	9.6	Unregistered	LUHSKC
7	F	16	2005	Renal carcinoma (L)	NS	CR	Alive	11.1	Registered	VUHSK
8	F	7	2005	Adrenocortical carcinoma (L)	NS	Progress	Died	2.2	Registered	VUHSK
9	M	4	2005	Intestinal hemangioendotelioma (L)	NS	Progress	Died	0.6	Registered	VUHSK
10	F	8	2005	Medullary thyroid carcinoma (L)	MEN2	Relapse	Died	11.8	Unregistered	LUHSKC
11	F	17	2006	Renal carcinoma (L)	NS	CR	Alive	13.6	Registered	LUHSKC
12	F	17	2006	Medullary thyroid carcinoma (L)	NS	Progress	Died	7.0	Registered	LUHSKC
13	F	17	2006	Pancreatic papillary carcinoma	NS	CR	Alive	13.5	Registered	VUHSK
14	M	11	2006	Intestinal neuroendocrine tumor (L)	NS	Progress	Died	0.7	Registered	VUHSK
15	F	15	2006	Renal carcinoma (L)	NS	CR	Alive	13.1	Registered	LUHSKC
16	F	0	2006	Adrenocortical carcinoma (A)	NS	Progress	Died	0.1	Registered	LUHSKC
17	F	17	2007	Adrenocortical carcinoma (L)	NS	Relapse	Died	8.9	Registered	LUHSKC
18	F	1	2007	Kaposi-like hemangioendothelioma (L)	NS	CR	Alive	12.1	Registered	VUHSK
19	F	15	2009	Ovarian carcinoma (L)	NS	CR	Alive	10.2	Registered	LUHSKC
20	M	6	2010	Renal carcinoma (L)	NS	CR	Alive	9.9	Unregistered	LUHSKC
21	M	15	2010	Follicular dendritic sarcoma (L)	NS	Progress	Died	0.4	Registered	VUHSK
22	F	16	2010	Ovarian carcinoma (A)	NS	Progress	Died	0.1	Registered	LUHSKC
23	F	10	2010	Carcinoma of upper lip (L)	NS	Relapse	Alive	7.0	Registered	VUHSK
24	M	14	2010	Adenocarcinoma of stomach (L)	NS	CR	Alive	9.2	Registered	VUHSK
25	F	0	2010	Adrenocortical carcinoma (L)	Li Fraumeni	2ndCa	Died	6.2	Registered	VUHSK
26	F	14	2011	Adrenocortical carcinoma (A)	NS	Relapse	Died	2.6	Registered	VUHSK
27	M	15	2012	Colorectal adenocarcinoma (L)	NS	Progress	Died	0.3	Registered	LUHSKC
28	M	12	2012	Adenocarcinoma of stomach (L)	NS	Progress	Died	0.1	Registered	LUHSKC
29	F	15	2012	Salivary gland carcinoma (L)	NS	CR	Alive	7.2	Unregistered	LUHSKC
30	M	17	2013	Pheochromocytoma (L)	VHL	CR	Alive	6.9	Registered	LUHSKC
31	M	0	2013	Rhabdoid tumor^1^ (L)	NS	TRM	Died	0.3	Registered	VUHSK
32	M	8	2013	Retroperitoneal DSRCT (A)	NS	Relapse	Died	2.1	Registered	VUHSK
33	M	14	2013	Skin melanoma (L)	NS	CR	Alive	6.7	Registered	VUHSK
34	F	9	2014	Medullary thyroid carcinoma (L)	MEN2	CR	Alive	5.9	Unregistered	LUHSKC
35	M	3	2014	Rhabdoid tumor^2^ (A)	NS	CR	Alive	5.7	Registered	VUHSK
36	M	0	2014	Rhabdoid tumor^3^ (L)	NS	Progress	Died	0.6	Registered	VUHSK
37	M	12	2015	Salivary gland carcinoma (L)	NS	CR	Alive	4.1	Unregistered	LUHSKC

2ndCa, the second cancer; A, advanced stage at diagnosis; CR, complete remission; DSRCT, desmoplastic small round cell tumor; F, female; L, local disease at diagnosis; LUHSKC, Lithuanian University of Health Sciences Kaunas Clinics; M, male; MEN2, multiple endocrine neoplasia type 2; NS, not specified; TRM, treatment related mortality; UPN, unique patient number in the study; VHL, von Hippel Lindau syndrome; VUHSK, Vilnius University Hospital Santaros Klinikos.

^1^Rhabdoid tumor of the thoracic wall.

^2^Rhabdoid tumor of multiple supradiaphragmatic lymph nodes and lung metastases.

^3^Rhabdoid tumor of right axillar soft tissue.

In five patients (13.5%), the underlying cancer predisposition syndromes were documented (Table 1). Thirty-one VRTs (83.8%) presented as a localized tumor, whereas in 6 (16.2%) cases an advanced stage with local or distant metastases was documented at diagnosis. Disease relapse or progression was the main cause that compromised the cure: 17 (45.9%) children died due to resistant malignancy accounting for 85% of all deaths. Two patients (UPN 6 and 25 in the Table 1) developed a second cancer that was the cause of death. Thus, 19 out of 20 (95%) patients who did not survive succumbed to progression of primary tumor or secondary malignancy. One infant (UPN 31, Table 1) developed a fatal cytomegalovirus-associated pneumonitis. At the time of evaluation, 17 out of 37 children (45.9%) remained in complete remission.

### Registration at the Lithuanian cancer registry

All 37 VRT entities included in the study were confirmed by pathology evaluation. The tumors were assigned a specific morphology and behavior code as well as an appropriate ICD-10-AM topography code C00–C96. All patients were eligible for registration at the LCR at diagnosis.

The cross-check of the cases identified in the institutional databases with the LCR data revealed that six out of 37 patients (16.2%) were not reported at the time of diagnosis (Table 1). All of them were diagnosed at LUHSKC and comprised 40% (6/15) of pediatric VRTs treated at the center. The missing cases included salivary gland and thyroid carcinoma (2 cases each), and a single case of adrenocortical carcinoma and uterine adenosarcoma (Fig. 1).

### Treatment outcomes

**Figure 2:**
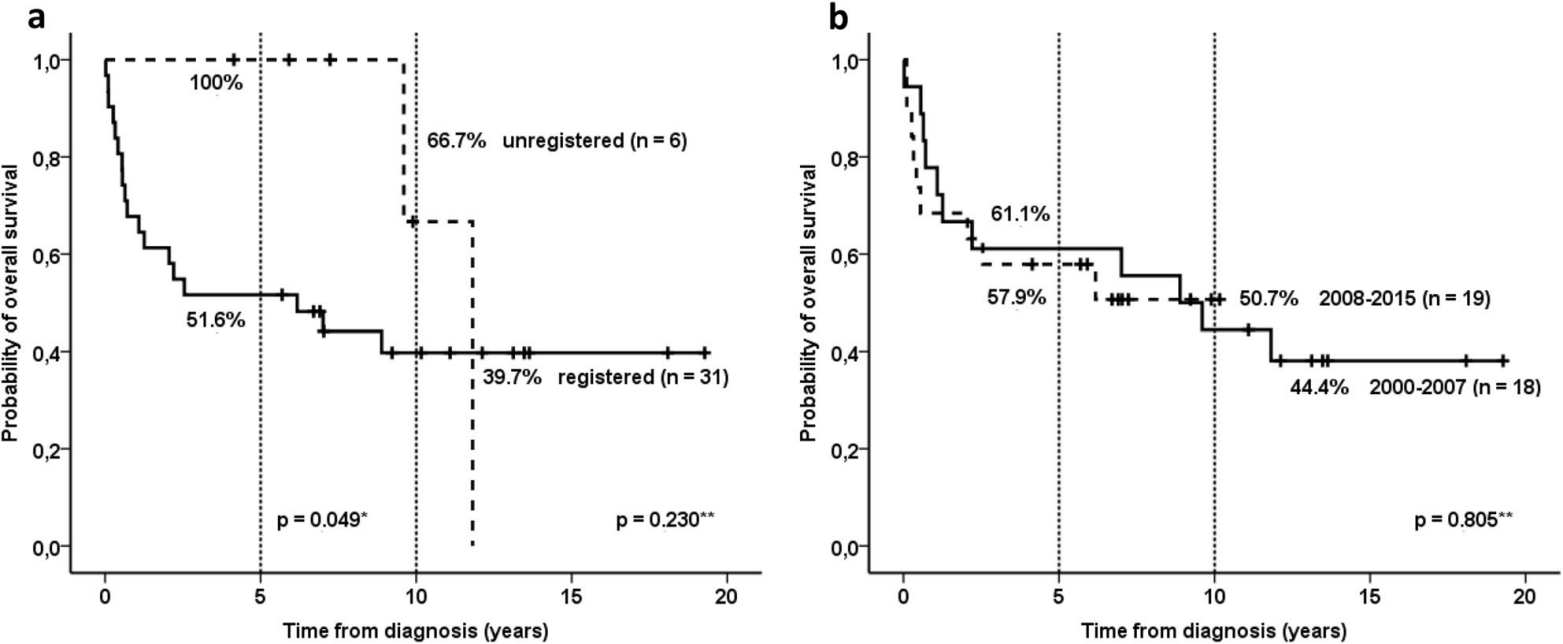
Probability of overall survival according to registration status (**a**) and treatment period (**b**). *Log-rank test for data censored at 5 years; **Log-rank test for data censored at the last follow-up. The survival probabilities were calculated using IBM SPSS Statistics 27.0 (https://www.ibm.com/partnerworld/bpdirectory/partner/6q81h/insight-solutions/6q81i/).

The overall survival estimate at 5 years of the entire cohort (including registered and unregistered patients) diagnosed with VRTs at the age of 0–18 years was 59.5% (Table 2). However, the same estimate calculated only for the registered patients was by 8 percent inferior—51.6%. The difference increased up to 10% when calculated only for children aged 0–14 years at diagnosis—52.2% versus 42.1% (Table 2). The difference at 1 year between the entire and the registered groups remained significant: 73.0% versus 67.7% for the age group of 0–18 years and 73.9% versus 68.4% for the group of 0–14 years, respectively (Table 2). In long-term perspective, the survival rates continued to decline beyond 5 years after diagnosis (Fig. 2): survival estimates at 10 years were lower than at 5 years retaining the difference between the entire and the registered cohorts: 44.3% versus 39.7% for the age group of 0–18 years and 46.4% versus 36.1% for the group of 0–14 years, respectively (Table 2).

**Table 2:** Survival estimates at 1-year, 5-years and 10-years for children diagnosed with a VRT at the age of 0–14 and 0–18 years: the impact of the registration completeness on the calculated rates. The estimates were calculated using STATA IC 11.0 (StataCorp LP). Licence number 30110538755. https://www.stata.com/.

Overall survival, alive % (95% CI*)	0–18 years		0–14 years	
	All patients (n = 37)	Registered only (n = 31)	All patients (n = 23)	Registered only (n = 19)
At 1 year	73.0 (55.6–84.4)	67.7 (48.3–81.2)	73.9 (50.9–87.4)	68.4 (42.8–84.4)
At 5 years	59.5 (42.0–73.2)	51.6 (33.0–67.4)	52.2 (30.5–70.0)	42.1 (20.4–62.5)
At 10 years	44.3 (26.6–60.7)	39.7 (22.1–56.8)	46.4 (25.0–65.4)	36.1 (15.7–57.1)

*CI—confidence interval.

The probability of OS_5y_ of the unregistered patients was significantly higher than the one of the registered cohort (100% vs 51.6%; *p* = 0.049, Fig. 2a). However, in a longer follow-up beyond 5 years from diagnosis the difference became insignificant due to late cancer-related deaths in both cohorts—the probability of OS_10y_ was 66.7% for unregistered patients as compared to 39.7% for the registered cohort (*p* = 0.230). The median follow-up time of the respective groups were 6.6 years (range 4.1–9.9 [IQR 4.6–9.2]) and 11.1 years (range 5.7–19.3 [IQR 6.0–13.6]). We could not document any improvement in the survival rates over the analyzed 16-year-period. The probability of overall survival for patients treated in 2000–2007 and 2008–2015 differed neither at five (61.1% vs 57.9%, respectively), nor at 10 years (44.4% vs 50.7%, respectively, *p* = 0.805, Fig. 2b). The median follow-up time was 13.5 (range 11.1–19.3 [IQR 12.1–18.1]) and 7.0 (range 4.1–10.2 [IQR 5.9–9.4]) years for the first and the second treatment periods, respectively.

## Discussion

Our study aimed to evaluate the quality of registration and treatment outcomes of pediatric VRTs in Lithuania—a small Eastern European country of 2.92 million inhabitants and 524.5 thousand children below 18 years of age (as reported in 2015, https://osp.stat.gov.lt). Overall, 37 VRT cases of 18 different histologic types were identified over 16 years with the median of 1.5 new cases per year. This means that each Lithuanian pediatric oncology center encountered one-two new VRT cases per year. The small numbers render impossible accumulation of sufficient expertise to deal with extremely rare pediatric malignancies. Therefore, close national and international collaboration e.g. within tumor-specific expert groups and/or the European Reference Network for Paediatric Oncology (ERN PaedCan) is crucial to ensure the best care and cure.

Inconsistency in the national regulatory requirements could partially underpin a substantial percentage of unregistered tumors in our study (16.2%). There was a formal obligation for health care providers to report every new cancer case to the LCR. However, the legal status of the registry was not appropriately formalized. This resulted in different interpretation of the reporting obligation and restriction in data flow. All unregistered cases came from one of two pediatric oncology centers and reflected institutional policy with regard to data sharing: in 2013–2016 LUHSKC refused to provide notifications on new cancer cases to LCR (both adult and pediatric). The data flow was fully restored in 2019. Different interpretation of regulatory requirements for data reporting contributed to the data incompleteness in the Estonian Cancer Registry^10^. However, the majority of the unregistered cases in our cohort stemmed from earlier period that could reflect the lack of institutional “registration culture” and awareness of its crucial impact on data interpretation^13^. Most of the missing data were entered to the LCR database retrospectively.

As listed in the Table 1, all unregistered tumors were localized at diagnosis (unfortunately, data on exact stage at presentation were not available). One could speculate that a potential contributing factor to the under-reporting of -non-advanced malignancies could be insufficient awareness of surgeons (who used to be the first to encounter a VRT in children and adolescents) about the importance of meticulous registration of every pediatric cancer case. Presumably, pediatric oncologists were not involved in these patients’ initial care and management. Several studies have shown that multidisciplinary teamwork positively affects the diagnosis, management and quality of care in cancer patients^14,15^. Thus, regular tumor boards, including virtual tumor boards as well as international collaborations, should be regarded as a standard of care in the management of childhood cancers^16,17^. Improvement in multidisciplinary collaboration between pediatric oncologists, surgeons, and cancer registrars could ensure completeness of registration and data reliability.

As expected, the OS_5y_ of the unregistered patients was significantly higher than the one of the registered cohort (100% vs 51.6%). The OS_5y_calculated for the registered patients at the age of 0–14 years—a common parameter used across studies to compare treatment outcomes and cancer incidence^1,7–10^—increased from 42.1 to 52.2% when missing cases were included. Given the extreme rarity of VRTs, accurate reporting of each case to cancer registries is crucial for reliable calculation of treatment outcomes. Insufficient registration of pediatric cancers was previously reported for more common childhood cancers, e.g. for CNS tumors. The analysis of the survival of European children based on the national population-based cancer registry data highlighted incomplete registration of non-malignant entities in many countries and, as a consequence, a lower overall survival^8^. The recent international survey focused on the rate of pleuropulmonary blastoma in Europe also demonstrated lower than estimated number of reported cases in Eastern/Central European countries^18^. A population-based quality study carried out in Estonia figured out a significant number of under-reported cases of childhood cancer that augmented the 5-year survival from 70 to 76% for children treated in 2010–2014^10^. Thus, completeness of the registration should be improved across Europe.

The main limitation of our population-based study was small number of patients. The enrolled cohort included only tumors that matched the definition of a VRT. Data collection process identified additional unregistered childhood cancer cases (some of them fatal), however most of them were CNS tumors of benign histology or non-malignant borderline neoplasms that are not reported to cancer registries. Thus, the final cohort was purified to avoid selection bias. Although the sample size was small and unbalanced, it could demonstrate significant impact of incomplete registration on outcome interpretation.

Another limitation of our research was its retrospective nature. The cohort included patients treated in early 2000ies that did not allow us to verify relevant parameters (e.g. details on initial stage at presentation, adverse events, treatment etc.) due to limited data availability. The overall survival rate in our VRT cohort was inferior (the total OS_5y_ was 59.5%) as compared to the average 80% cure rate of more common childhood cancers. Similar outcomes were reported for adults in a population-based Surveillance of Rare Cancers in Europe (RARECARE) project^19^—rare cancers displayed lower survival rate (47%) than the common cancers (65%). A scarce expertise due to the rarity of cases and absence of clinical trials were main contributors to the inferior survival. Pediatric tumor-specific studies likewise reported lower survival rate in rare malignancies, e.g. 55% for adrenocortical carcinoma^20^, 60% for rhabdoid tumor^21^, although some localized pediatric VRTs (e.g. thyroid and salivary gland carcinomas) can achieve a survival over 90%^22,23^.

The lack of improvement in survival rate over time (the OS_5y_ was 61.1% in 2000–2007 vs 57.9% in 2008–2015) was rather unexpected since national population-based studies on leukemia^24,25^ and single-center reports on solid tumors^26–28^ demonstrated significant improvement in overall survival over the last two decades. In our VRT cohort, the disease recurrence or development of a second malignancy were responsible for 95% of deaths (in 19 out of 20 cases). Drug-resistant cancer remains the main challenge for pediatric oncology community: a recent review of the Surveillance, Epidemiology, and End Results (SEER) database demonstrated little improvement in treatment outcome for specific cancer types, mostly considered as VRT, diagnosed in adolescents or young adults during 1975–2011^29^. Of note, in our study a cancer predisposition syndrome was documented in five of 37 patients (Table 1). Impaired somatic host genome could be implicated in development of drug-resistance or secondary malignancy^30,31^. Our results suggest that the diagnosis of a VRT in a child should prompt a search for possible hereditary genetic susceptibility syndrome.

Disease recurrence was found to compromise the cure beyond 5 years, which is a usually used cut-off for long-term remission. The survival estimates at 10 years were lower than those at 5 years calculated with regard to various aspects: for different age groups, completeness of the registration, for registered and unregistered cohorts as well as for two treatment periods. Late events were related exclusively to disease progression or second malignancy. A similar decline in survival rates at 5 and 10 years after treatment (from 61.1 to 56.8%, respectively) was demonstrated in a large cohort of 1269 children treated for cancer in Lithuania between 1982 and 2011^32^. The above mentioned analysis of SEER database reported a substantial number of the disease-related adverse events occurring between 5 and 10 years post-therapy in patients diagnosed with rare cancers at the age of 15–39 years^29^. Given the fact that 75% of VRTs develop between the ages of 15 to 19 years^3–5^, a longer follow-up for potential disease recurrence beyond 5 years from diagnosis is warranted.

Our results clearly demonstrated that timely and complete registration of VRTs must be ensured for accurate statistical analysis and data evaluation. In addition to mandatory national reporting regulations, an ongoing European PARTNER (Pediatric Rare Tumor Network—European Registry) project supported by ERN PaedCan aims to create a pan-European system that should enhance international communications between members of the European Union^33^. Especially, the Work Package 7 (WP7) of the PARTNER project aims to improve the quality of care of patients with VRT Low Health Expenditure Average Rate (LHEAR) countries. This is achieved by combining national registries focused on VRTs and creating registries for countries that do not have one, as well as linking these registries with virtual consultation systems (https://webgate.ec.europa.eu). The undertaken actions will certainly strengthen registration at the national levels.

## Conclusions

Incomplete registration of VRTs in cancer registry can significantly affect epidemiologic and outcome data. Regular verification of the pediatric cancer cases could ensure data quality and completeness of registration. Based on our results we would strongly advocate for an active collaboration between pediatric oncology centers and national cancer registries to prevent important deviation in statistical analysis and calculation of survival data. International cooperation within EU projects and ERN PaedCan network may improve diagnostics, management and registration of pediatric VRTs across European countries.

## Supplementary Information

Supplementary Table.

Supplementary Information.

## Data Availability

The datasets generated and analyzed during the current study are not publicly available due to data protection and privacy but are available from the corresponding author on reasonable request.

## Abbreviations

CNS: Central nervous system
ExPeRT: European Cooperative Study Group for Pediatric Rare Tumours
IQR: Interquartile range
LCR: Lithuanian Cancer Registry
LUHSKC: Lithuanian University of Health Sciences Kaunas Clinics
OS_5y_: Overall survival at 5 years
OS_10y_: Overall survival at 10 years
VRT: Very rare tumor
VUHSK: Vilnius University Hospital Santaros Klinikos
