# Supplementary material for: The impact of incomplete registration on survival rate of children with very rare tumors
Source: Sci Rep. 2021 Jul 7;11:14066. doi: 10.1038/s41598-021-93670-2 (PMC8263601; doi:10.1038/s41598-021-93670-2)
Supplement: Supplementary file 1 — Supplementary Table. [file 41598_2021_93670_MOESM1_ESM.docx]

**Supplementary Table S**. Baseline characteristics of the study patients according to the treating pediatric oncology center (n = 37).

|  | VUHSK  (n = 22) | LUHSKC  (n = 15) | Totally  (n = 37) |
| --- | --- | --- | --- |
| VRTs diagnosed in | 2000 – 2013 | 2005 – 2015 | 2000 – 2015 |
| Age at diagnosis, years  Median* (min-max)  IQR | 10.5 (0-17)  2.8-14.3 | 15.0 (0-17)  9.0-17.0 | 12 (0 - 17)  6.5-15.5 |
| Sex, number (%)  Boys  Girls | 11 (50)  11 (50) | 5 (33.3)  10 (66.7) | 16 (43.2)  21 (56.8) |
| VRT type, number (%)  Adrenocortical carcinoma  Hemangioendothelioma  Renal carcinoma  Thyroid cancer  Rhabdoid tumor  Pheochromocytoma  Gastric adenocarcinoma  Salivary gland carcinoma  Ovarian cancer  Colorectal carcinoma  Desmoplastic small round cell tumor  Follicular dendritic cell sarcoma  Intestinal neuroendocrine carcinoma  Lip carcinoma  Lung carcinoma  Melanoma  Pancreatic carcinoma  Uterine adenosarcoma | 4 (18.2)  4 (18.2)  2 (9.1)  -  3 (13.6)  1 (4.5)  1 (4.5)  -  -  -  1 (4.5)  1 (4.5)  1 (4.5)  1 (4.5)  1 (4.5)  1 (4.5)  1 (4.5)  - | 3 (20)  -  1 (6.7)  3 (20)  -  1 (6.7)  1 (6.7)  2 (13.3)  2 (13.3)  1 (6.7)  -  -  -  -  -  -  -  1 (6.7) | 7 (18.9)  4 (10.8)  3 (8.1)  3 (8.1)  3 (8.1)  2 (5.4)  2 (5.4)  2 (5.4)  2 (5.4)  1 (2.7)  1 (2.7)  1 (2.7)  1 (2.7)  1 (2.7)  1 (2.7)  1 (2.7)  1 (2.7)  1 (2.7) |
| Disease dissemination at diagnosis, number (%)  Local  Metastatic | 18 (81.8)  4 (18.2) | 13 (86.7)  2 (13.3) | 31 (83.8)  6 (16.2) |
| CR**, number (%)  CR  Event*** | 9 (40.9)  13 (59.1) | 7 (46.7)  8 (53.3) | 16 (43.2)  21 (26.8) |
| Survival status**, number (%)  Alive  Dead | 10 (45.5)  12 (54.5) | 7 (46.7)  8 (53.3) | 17 (45.9)  20 (54.1) |
| Follow-up, years from diagnosis  Median (min-max)  IQR | 11.8 (5.7-19.3)  6.9-14.8 | 7.2 (4.1-13.1)  5.9-10.2 | 9.9 (4.1-19.3)  6.8-13.3 |

*p = 0.065 (Mann-Whitney test for median age difference)

** status at the data evaluation time-point

***event was defined as treatment toxicity-related death, relapse or disease progression, second malignancy whichever occurred first

Abbreviations: CR – complete remission; IQR – interquartile range; LUHSKC – Lithuanian University of Health Sciences Kaunas Clinics; VRT – very rare tumor; VUHSK – Vilnius University Hospital Santaros Klinikos.
