# Supplementary figures and images for: The impact of incomplete registration on survival rate of children with very rare tumors
Source: Sci Rep. 2021 Jul 7;11:14066. doi: 10.1038/s41598-021-93670-2 (PMC8263601; doi:10.1038/s41598-021-93670-2)

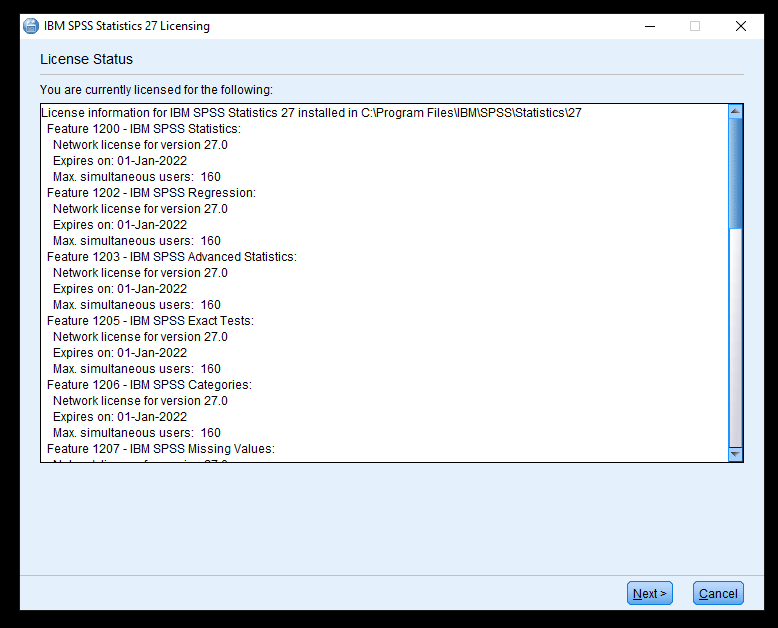

Supplement: Supplementary file 2 — Supplementary Information. [file 41598_2021_93670_MOESM2_ESM.png]
